# Serum level of soluble interleukin‐2 receptor is positively correlated with metabolic tumor volume on ^18^F‐FDG PET/CT in newly diagnosed patients with diffuse large B‐cell lymphoma

**DOI:** 10.1002/cam4.1973

**Published:** 2019-02-20

**Authors:** Hajime Senjo, Minoru Kanaya, Koh Izumiyama, Koichiro Minauchi, Kenji Hirata, Akio Mori, Makoto Saito, Masanori Tanaka, Hiroaki Iijima, Eriko Tsukamoto, Kazuo Itoh, Shuichi Ota, Masanobu Morioka, Daigo Hashimoto, Takanori Teshima, North Japan Hematology Study Group (NJHSG)

**Affiliations:** ^1^ Department of Hematology Aiiku Hospital Sapporo Japan; ^2^ Department of Hematology Sapporo Hokuyu Hospital Sapporo Japan; ^3^ Department of Nuclear Medicine, Graduate School of Medicine Hokkaido University Sapporo Japan; ^4^ Clinical Research and Medical Innovation Center Hokkaido University Hospital Sapporo Japan; ^5^ Department of Radiology Central CI Clinic Sapporo Japan; ^6^ Department of Radiology Keiyukai Sapporo Hospital Sapporo Japan; ^7^ Department of Hematology, Faculty of Medicine Hokkaido University Sapporo Japan

**Keywords:** diffuse large B‐cell lymphoma, soluble interleukin‐2 receptor, total metabolic tumor volume

## Abstract

Diffuse large B‐cell lymphoma (DLBCL) is the most frequent subtype of non‐Hodgkin lymphoma. High total metabolic tumor volume (TMTV) calculated using ^18^F‐FDG PET/CT images at diagnosis predicts poor prognosis of patients with DLBCL. However, high cost and poor access to the imaging facilities hamper wider use of ^18^F‐FDG PET/CT. In order to explore a surrogate marker for TMTV, we evaluated the correlation between the serum levels of soluble interleukin‐2 receptor (sIL‐2R) and TMTV in 64 patients with DLBCL, and the results were verified in an independent validation cohort of 86 patients. Serum levels of sIL‐2R were significantly correlated with TMTV. ROC analysis revealed that the cutoff value of TMTV ≥150 cm^3^ or sIL‐2R ≥ 1300 U/mL could predict failure to achieve EFS24 with areas under the curve (AUC) 0.706 and 0.758, respectively. Each of TMTV ≥150 cm^3^ and sIL‐2R ≥1300 U/mL was significantly associated with worse 5‐year overall survival and event‐free survival. Importantly, each of sIL‐2R <1300 U/mL or TMTV <150 cm^3^ identified patients with favorable prognosis among NCCN‐IPI high‐intermediate and high‐risk group. Serum level of sIL‐2R represents a convenient surrogate marker to estimate metabolic tumor burden measured by ^18^F‐FDG PET/CT that can predict treatment outcomes of patients with DLBCL.

## 1 INTRODUCTION

Diffuse large B‐cell lymphoma (DLBCL) is the most common subtype of malignant lymphoma, accounting for 30%‐40% of non‐Hodgkin lymphoma.^1^ Although rituximab combined with cyclophosphamide, doxorubicin, vincristine, and prednisone (R‐CHOP) has led to a remarkable improvement in the treatment of DLBCL patients,^2^ considerable proportion of the patients fail to cure.^3^ To date, many prognostic factors are advocated, including patient factors: age, performance status (PS),^3^ Charlson Comorbidity Index,^4^ tumor burden; LDH,^5^ clinical stage,^5^ and biological features of tumor cells; and germinal center B‐cell (GCB) type or non‐GCB type,^6^, ^7^ CD5‐positivity,^8^ status of Epstein‐Barr virus,^9^ and double‐hit lymphoma.^10^ These factors should be considered comprehensively at diagnosis to estimate the prognosis of the patients with DLBCL.

^18^F‐FDG PET/CT is widely performed at initial staging in DLBCL patients. High total metabolic tumor volume (TMTV) calculated using ^18^F‐FDG PET/CT images at diagnosis is predictive of poor prognosis of DLBCL, follicular lymphoma (FL), and peripheral T‐cell lymphoma.^11^, ^12^, ^13^, ^14^ However, not all patients can undergo ^18^F‐FDG PET/CT due to various reasons, including high cost, poor access to the imaging facilities, and urgent requirement for treatment initiation, encouraging us to explore a surrogate marker for metabolic tumor volumes. Several previous studies have shown that serum level of sIL‐2R is a prognostic biomarker of DLBCL.^15^, ^16^ In the current study, we retrospectively evaluated the correlation between the serum levels of sIL‐2R and TMTV based on ^18^F‐FDG PET/CT images at diagnosis and compared the role of these parameters as prognostic biomarker in newly diagnosed DLBCL.

## 2 PATIENTS AND METHODS

### 2.1 Patients

In the training cohort, we reviewed the medical records of 64 consecutive adult patients with DLBCL newly diagnosed according to the 4th Edition of World Health Organization (WHO) classification at Aiiku Hospital from 2008 to 2014. In the validation cohort, we reviewed the medical records of 86 patients with DLBCL newly diagnosed at Sapporo Hokuyu Hospital from 2008 to 2013. All patients in the training cohort underwent ^18^F‐FDG PET/CT at Central CI clinic (Sapporo, Japan) with PET/CT device (Discovery ST Elite^®^; GE Healthcare, Tokyo, Japan, or GEMINI^®^; Philips, Tokyo, Japan), and those in the validation cohort underwent ^18^F‐FDG PET/CT at Keiyukai Sapporo Hospital (Sapporo, Japan), with PET/CT device (GEMINI^®^ GXL; Philips, Tokyo, Japan) before initiation of chemotherapy. We did not cross calibrate cameras for PET/CT at these two facilities. Serum levels of sIL‐2R were measured using chemiluminescent enzyme immunoassay (CLIA; STACIA^®^; LSI Medience, Tokyo, Japan) in the training cohort, while those were measured using enzyme‐linked immunosorbent assay (ELISA; IL‐2Rtest^®^; BML, Tokyo, Japan) in the validation cohort. The study procedures were in accordance with the Helsinki Declaration and institutional ethical guidelines, conducted under the auspices of the institutional ethics committee, and approved by the institutional review boards of each institute. Clinical stage was determined according to the Ann Arbor staging system, and treatment response was evaluated according to the International Workshop criteria.^17^ NCCN‐IPI scores were calculated as previously described.^5^

### 2.2 PET/CT parameters

Standardized uptake value (SUV) was calculated as [tissue radioactivity concentration (Bq/mL)] × [body weight (g)]/[injected radioactivity (Bq)]. TMTV was defined as the volume of lymphoma visualized on PET/CT scans with SUV greater than or equal to an absolute threshold of 4.0, as previously described.^18^ SUV computer‐aided analysis of PET/CT images for TMTV calculations was performed using Metavol (Hokkaido University, Sapporo, Japan, http://www.metavol.org/home),^19^ with exclusion of physiological accumulation including urinary, myocardial, and brain FDG uptake. Nodular or heterogeneous uptake in the bone marrow was included as tumor involvement based on radiologist's interpretation, while diffuse uptake was considered as physiological uptake. All quantitative parameters were retrospectively measured by a nuclear medicine physician (ET) in a blinded fashion.

### 2.3 Statistical analysis

Overall survival (OS) was calculated from the day of diagnosis until death or last follow‐up. Event‐free survival (EFS) was defined as time from diagnosis to disease progression, relapse after response, death, or last follow‐up. The probabilities of OS and EFS were estimated using a Kaplan–Meier method, and differences between patient groups were analyzed using the log‐rank test. The baseline patient characteristics were tabulated to check imbalance in the demographic information. The risk factors at diagnosis for OS or EFS were evaluated by multivariate Cox regression using stepwise variable selection. Analysis of contingency data of sIL‐2R and TMTV was carried out using Fisher's exact test (categorical variables) and Mann‐Whitney *U* test (continuous variables). Youden Index was calculated to determine optimal cutoff value of these parameters in receiver operating curve (ROC) analysis with regarding failure in achievement of 2‐year EFS (EFS24) 20, 21 as positive finding. The correlation between sIL‐2R and TMTV was assessed by using Pearson's product‐moment correlation coefficient, respectively. All *P*‐values were 2‐sided, and a *P*‐value of 0.05 was used as the cutoff for statistical significance. All the statistical analyses were performed with the EZR (http://www.jichi.ac.jp/saitama-sct/SaitamaHP.files/statmedEN.html).^22^

## 3 RESULTS

### 3.1 Patient characteristics

Baseline patient characteristics were listed in Table 1. In the training cohort, the median patient age at diagnosis was 74 years, ranging from 33 to 86 years. PS was 2 or greater in 30% of the patients, and 81% of the patients had stage III or IV. Sixty‐six percent of the patients had extranodal involvement, including bone marrow (17%), and 58% presented with B‐symptoms. Sixty‐seven percent of the patients had elevated serum LDH value than normal level. For NCCN‐IPI scores, 24%, 31%, and 45% of the patients were classified as Low or Low‐intermediate (Low‐int) risk group, High‐intermediate (High‐int) risk group, and High‐risk group, respectively.

**Table 1:** Patient characteristics

Characteristics	Training cohort No. (%)	Validation cohort No. (%)	*P* value
Sex (male/female)	31/33	44/42	0.512
Age (median y, range)	74 (33‐86)	71 (24‐90)	0.019
ECOG Performance status			<0.01
0, 1	45 (70)	81 (94)	
≥2	19 (30)	5 (6)	
Stage			<0.01
I, II	12 (19)	54 (63)	
III	13 (20)	11 (13)	
IV	39 (61)	21 (24)	
Extranodal sites			0.281
0	33 (34)	60 (70)	
≥1	42 (66)	26 (30)	
Bone marrow involvement			0.541
Yes	11 (17)	10 (12)	
No	53 (83)	76 (88)	
B symptoms			0.330
Yes	29 (58)	12 (14)	
No	35 (42)	74 (86)	
LDH			0.285
≤Normal	21 (33)	45 (52)	
≥Normal	43 (67)	41 (48)	
sIL‐2R			0.409
Median (range)	1735 (243‐43 700) U/mL	1274 (200‐39 798) U/mL	
<1300	27 (42)	40 (47)	
≥1300	37 (58)	46 (53)	
TMTV			0.411
Median (range)	236.32 (76.62‐677.09) cm^3^	167.2305 (4.61‐5445.50) cm^3^	
<150	26 (41)	44 (51)	
≥150	38 (59)	42 (49)	
NCCN‐IPI			0.014
Low, Low‐int	15 (24)	47 (55)	
High‐int	20 (31)	33 (38)	
High	29 (45)	6 (7)	
Treatment			<0.01
R‐CHOP	27(42)	81(94)	
R‐THP‐COP	36(56)	5(6)	
R‐CVP	1(2)	0(0)	
Outcome			
CR	42 (66)	69 (80)	0.0594
PR	7(11)	2(2)	0.0378
Residual disease	18 (28)	18 (21)	0.338
Relapse	16 (25)	14 (16)	0.218
Death from disease	12 (19)	5 (6)	0.0184
Treatment‐related death	11 (17)	8 (9)	0.214
Death from other reasons	5 (8)	2 (2)	0.134

CR, complete remission; ECOG, Eastern Cooperative Oncology Group; LDH, lactate dehydrogenase; NCCN‐IPI, National Comprehensive Cancer Network‐International Prognostic Index; PR, partial remission; residual disease includes the patient with PR, stable disease, and progressive disease.; R‐CHOP, rituximab, cyclophosphamide, doxorubicin, vincristine, and prednisone; R‐CVP, rituximab, cyclophosphamide, vincristine, and prednisolone; R‐THP‐COP, rituximab, therarubicin, cyclophosphamide, vincristine, and prednisone; sIL‐2R, soluble interleukin‐2 receptor; TMTV, total metabolic tumor volume.

### 3.2 Treatment and outcome

**Figure 1:**
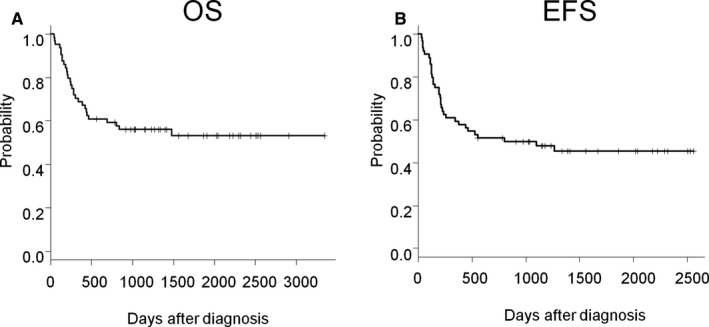
Kaplan‐Meier plots of OS (A) and EFS (B) of patients in the training cohort (n = 64)

Patients in the training cohort were initially treated with R‐CHOP or R‐CHOP‐like chemotherapies (R‐THP‐COP: rituximab, pirarubicin, cyclophosphamide, vincristine, and prednisone; R‐CVP: cyclophosphamide, vincristine, and prednisolone).^23^, ^24^ Nineteen percent of the patients received additional involved field radiation therapy following completion of chemotherapy. Overall response was 77% (CR + CRu: 66%, PR: 11%). With a median follow‐up period of 32.8 months, ranging from 1.4 to 111.5 months, an estimated OS rate was 53.1% (95% CI: 39.5%‐64.9%) and EFS rate was 45.4% (95% CI: 32.5%‐57.4%) at 5 years (Figure 1).

### 3.3 sIL‐2R levels correlated with TMTV

**Figure 2:**
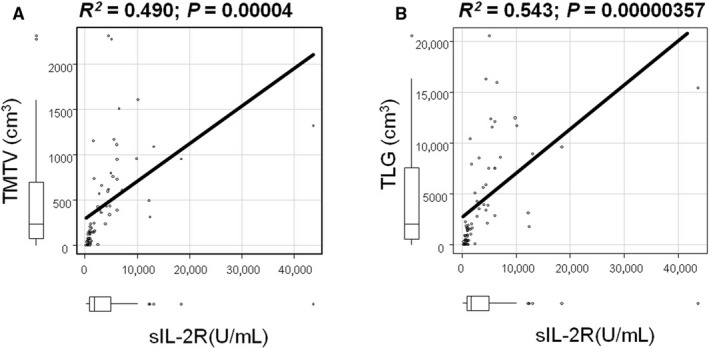
Correlation of sIL‐2R level with TMTV. In the training population (n = 64), positive correlation between sIL‐2R and TMTV (Pearson *R* ^2^ = 0.490; *P* = 0.00004) (A) is shown. Positive correlation between sIL‐2R and TMTV (Pearson *R* ^2^ = 0.461; *P* = 0.00000631) (B) in the validation cohort (n = 86) is shown

Pearson's correlation tests demonstrated highly significant positive correlation between sIL‐2R and TMTV (*R* ^2^ = 0.490; *P* = 0.00004, Figure 2A).

### 3.4 Quantitative PET parameters as prognostic biomarkers

**Figure 3:**
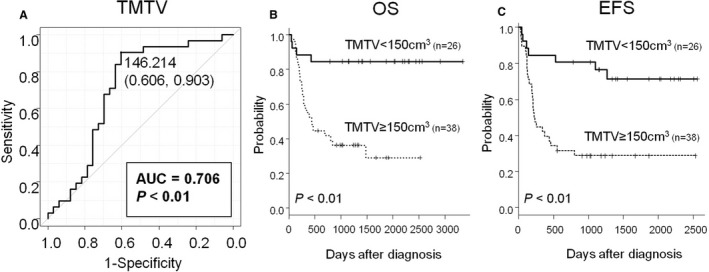
ROC according to TMTV. In the training cohort, with regarding failure in achievement of 2‐year EFS as positive finding, ROC according to TMTV (A) is shown. Kaplan‐Meier plots of OS (B) and EFS (C) according to TMTV is shown (n = 64)

In the training population, the mean TMTV was 460.45 cm^3^ (median 236.32 cm^3^, 25th‐75th percentiles 76.62‐677.09 cm^3^). ROC analysis revealed that the cutoff value of TMTV <150 cm^3^ could predict achievement of EFS24 (Figure 3A). AUC was 0.706 (95% CI: 0.570‐0.841; *P* = 0.00288). The 150 cm^3^ cutoff value for TMTV had a sensitivity and a specificity of 60.6% and 90.3%, respectively, for achievement of EFS24. OS and EFS were significantly lower in patients with TMTV ≥150 cm^3^ than in those with less than 150cm^3^ (5‐year OS; 84.0% vs 29.1%, *P = *0.000194, 5‐year EFS; 71.4% vs 28.7%, *P = *0.000384 (Figure 3B, C).

### 3.5 sIL‐2R level at diagnosis as a prognostic biomarker

**Figure 4:**
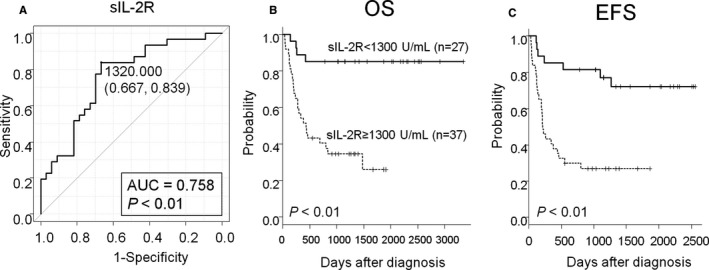
ROC according to sIL‐2R With regarding failure in achievement of 2‐y EFS as positive finding, a ROC according to sIL‐2R (A) is shown. Kaplan‐Meier plots of OS (B) and EFS (C) according to sIL‐2R were shown

In the training cohort, the median serum sIL‐2R level at diagnosis was 1735 U/mL, ranging from 243 to 43 700 U/mL. ROC analysis revealed that the cutoff value of sIL‐2R <1300 U/mL could predict achievement of EFS24 (Figure 4A). AUC was 0.758 (95% CI: 0.638‐0.877; *P* = 0.0000233). The 1300 U/mL cutoff value for sIL‐2R had a sensitivity and a specificity of 66.7% and 83.9%, respectively, for achievement of EFS24. Kaplan–Meier curves showed that sIL‐2R ≥1300 U/mL was a strong prognostic factor both for worse OS and EFS (5‐year OS; 85.2% vs 25.9%, *P = *0.000035, 5‐year EFS; 72.0% vs 26.8%, *P = *0.000076; Figure 4B, C).

### 3.6 Univariate and multivariate analyses of clinical prognostic factors

NCCN‐IPI was predictive for 5‐year OS (NCCN‐IPI: Low/Low‐Int, 93.3%; High‐Int, 59.2%; High, 23.0%, *P* = 0.000147) and 5‐year EFS (NCCN‐IPI: Low/Low‐Int, 83.0%; High‐Int, 54.0%; High, 23.0%, *P* = 0.000307; Figure S1). We analyzed various prognostic factors for OS and EFS (Table 2). In a univariate analysis, B symptom, LDH, sIL‐2R, and TMTV were associated with poor 5‐year OS; B symptom, LDH, PS, sIL‐2R, and TMTV were identified as poor prognostic factors for 5‐year EFS. We therefore performed multivariate analysis that included sIL‐2R and all factors in NCCN‐IPI; age, LDH, clinical stage, ECOG PS, and major organ involvement.^5^ Although serum levels of sIL‐2R were significantly higher in patients with PS ≥ 2, elevated LDH, or CS ≥ III, age and sIL‐2R were independently associated with poor 5‐year OS (age; HR, 4.44; 95% CI: 1.05‐18.7, *P* = 0.0424, sIL‐2R; HR, 4.45; 95% CI: 1.04‐19.1, *P* = 0.0444; Table 3 and Table S1). Another multivariate analysis that included TMTV and all factors for NCCN‐IPI demonstrated that TMTV was an only independent prognostic factor for 5‐year OS (HR: 3.87; 95% CI: 1.08‐13.8; log‐rank, *P* = 0.0373; Table 3). We performed an additional multivariate analysis including both TMTV and sIL‐2R We found that neither TMTV nor sIL‐2R persisted as an independent prognostic factor after this multivariate analysis, further confirming the strong correlations between TMTV and sIL‐2R (Table 3).

**Table 2:** Univariate analysis of the risk factors associated with 5‐y OS and EFS

Characteristics	Training cohort				Validation cohort			
	OS (%)	*P* value	EFS (%)	*P* Value	OS (%)	*P* value	EFS (%)	*P* Value
Sex		0.8		0.81		0.55		0.08
Male	51.7		44.8		11.4		52.3	
Female	57.1		48.6		16.7		71.4	
Age		0.53		0.10		0.07		0.64
<70	31.6		68.4		23.8		73.8	
≥70	22.2		44.4		22.0		68.2	
ECOG Performance Status		0.12		<0.01		1.00		0.32
<2	31.1		64.4		14.7		64.0	
≥2	10.5		21.1		9.1		45.5	
Stage		0.48		0.11		0.76		0.11
<Stage III	50.0		75.0		13.0		68.5	
≥Stage III	42.3		46.2		15.6		50.0	
Extranodal sites		0.07		0.07		0.50		0.81
0	40.9		68.0		11.7		60.0	
≥1	16.7		42.9		19.2		65.4	
Bone marrow involvement		0.52		0.21		0.63		0.50
Yes	50.0		38.2		20.0		50.0	
No	60.0		56.7		13.2		63.2	
B symptoms		<0.01		<0.01		0.67		1.00
Yes	31.0		27.6		16.7		58.3	
No	74.3		62.9		13.5		62.2	
LDH		0.03		<0.01		0.76		0.08
≤Normal	42.7		80.9		15.6		71.1	
>Normal	16.3		37.2		12.2		65.4	
sIL‐2R		<0.01		<0.01		0.21		<0.01
≤1300 U/mL	51.8		74.0		20.0		80.0	
>1300 U/mL	8.1		27.0		8.7		45.7	
TMTV		<0.01		<0.01		0.03		<0.01
≤150 cm^3^	24.0		80.8		22.7		77.3	
>150 cm^3^	7.9		31.6		4.8		59.4	

ECOG, Eastern Cooperative Oncology Group; EFS, event‐free survival; LDH, lactate dehydrogenase; OS, overall survival; sIL‐2R, soluble interleukin‐2 receptor; TMTV, total metabolic tumor volume.

Major organ involvement is defined as lymphomatous involvement in bone marrow, central nerve system, liver, gastrointestinal tract, or lung.

**Table 3:** Multivariate analysis of the risk factors associated with 5‐y OS

Characteristics	Training cohort			Validation cohort		
	Relative risk	95% CI	*P* value	Relative risk	95% CI	*P* value
Analysis including sIL‐2R						
Age ≥70	4.44	1.05‐18.7	0.04	2.03	1.19‐3.46	<0.01
ECOG Performance Status ≥2	3.26	0.83‐12.8	0.08	1.78	0.88‐3.62	0.11
LDH >Normal	2.40	0.45‐12.9	0.30	1.70	0.94‐3.08	0.08
Major organ involvement	1.01	0.22‐4.68	0.99	0.82	0.45‐1.52	0.53
Stage ≥III	0.75	0.09‐5.90	0.78	1.08	0.59‐2.02	0.79
sIL‐2R ≥1300 U/mL	4.45	1.04‐19.1	0.04	1.94	1.01‐3.72	0.05
Analysis including TMTV						
Age ≥70	2.47	0.93‐6.57	0.07	2.23	1.32‐3.75	<0.01
ECOG Performance Status ≥2	1.47	0.64‐3.38	0.36	1.96	0.95‐4.01	0.07
LDH >Normal	1.61	0.41‐6.22	0.48	2.14	1.20‐3.83	0.01
Major organ involvement	1.15	0.39‐3.32	0.79	0.67	0.37‐1.22	0.19
Stage ≥III	0.88	0.19‐4.04	0.87	1.10	0.62‐1.95	0.74
TMTV ≥150 cm^3^	3.87	1.08‐13.8	0.04	3.30	1.82‐6.00	<0.01
Analysis including sIL‐2R and TMTV						
Age ≥70	2.48	0.91‐6.72	0.07	2.24	0.87‐3.83	0.08
ECOG Performance Status ≥2	1.49	0.66‐3.39	0.34	1.87	0.75‐3.96	0.54
LDH >Normal	1.60	0.41‐6.20	0.49	1.79	0.79‐2.85	0.21
Major organ involvement	1.14	0.39‐3.29	0.81	1.02	0.38‐5.34	0.48
Stage ≥III	0.67	0.15‐3.04	0.61	1.10	0.57‐3.32	0.12
sIL‐2R ≥1300 U/mL	4.51	0.72‐28.5	0.11	2.02	0.74‐6.46	0.76
TMTV ≥150 cm^3^	1.23	0.17‐8.93	0.84	2.47	0.36‐5.48	0.13

ECOG, Eastern Cooperative Oncology Group; LDH; lactate dehydrogenase; OS, overall survival; sIL‐2R, soluble interleukin‐2 receptor.

**Figure 5:**
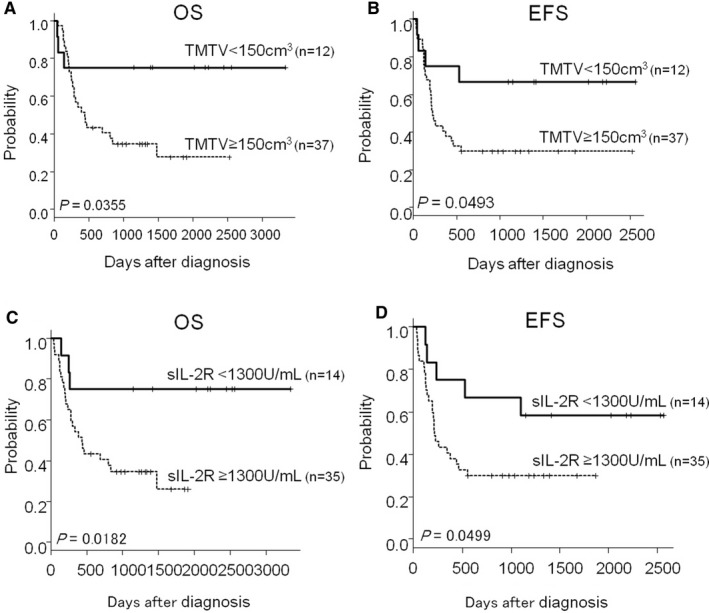
Impacts of TMTV and sIL‐2R levels in patients with High and High‐intermediate risk by NCCN‐IPI. Kaplan‐Meier plots of OS (A) and EFS (B) according to TMTV, OS (C) and EFS (D) according to sIL‐2R in patients with High risk (n = 29) and High‐intermediate risk (n = 20) stratified by NCCN‐IPI are shown

Subgroup analyses included the patients with NCCN‐IPI High‐Int and High (n = 49) demonstrated that the cutoff value of TMTV 150 cm^3^ stratified treatment outcomes in this poor prognostic group (5‐year OS; 75.0% vs 27.7%, *P = *0.0355, 5‐year EFS; 66.7% vs 29.7%, *P = *0.0493; Figure 5A, B). Similar results were obtained using the cutoff value of sIL‐2R 1300 U/mL (5‐year OS; 75.0% vs 25.9%, *P = *0.0182, 5‐year EFS; 58.3% vs 29.7%, *P = *0.0499; Figure 5C, D).

### 3.7 Validation of the results in the validation cohort

Finally, the prognostic impacts of serum levels of sIL‐2R and TMTV, and correlation between sIL‐2R and metabolic parameter were validated in the independent validation cohort composed of significantly younger patients with better PS, less advanced‐stage disease, and lower NCCN‐IPI scores than the training cohort (Table 1). The OS and EFS in this cohort were shown (Figure S2). In terms of TMTV and sIL‐2R, there were no significant differences between patients in the training cohort and the validation cohort (Table 1). Kaplan‐Meier curves showed that OS and EFS rates in patients with TMTV ≥150 cm^3^ were again lower than in those with TMTV <150 cm^3^ (5‐year OS; 87.0% vs 59.5%, *P = *0.016, 5‐year EFS; 72.8% vs 52.3%, *P = *0.0154; Figure S3A, B).

The median serum sIL‐2R level at diagnosis was 1274 U/mL, ranging from 200 to 39 798 U/mL. Kaplan‐Meier curves showed that sIL‐2R ≥1300 U/mL was a strong prognostic factor both for worse OS and EFS (5‐year OS; 86.3% vs 61.8%, *P = *0.0188, 5‐year EFS; 85.0% vs 46.8%, *P = *0.000413; Figure S3C, D). Pearson's correlation tests gave similar results that there were positive correlations between sIL‐2R and TMTV (*R* ^2^ = 0.461; *P* = 0.00000631; Figure 2B). In a univariate analysis, TMTV was associated with poor 5‐year OS, whereas sIL‐2R and TMTV were identified as poor prognostic factors for EFS (Table 2). In a multivariate analysis including sIL‐2R, age was an independent prognostic factor and there was a strong trend toward worse 5‐year OS in patients with higher sIL‐2R (Table 3). In another multivariate analysis including TMTV showed that age, LDH, and TMTV were independent prognostic factor for 5‐year OS (Table 3). Altogether, we could validate that both sIL‐2R and TMTV are promising prognostic biomarkers and there is a positive correlation between sIL‐2R and TMTV, suggesting that sIL‐2R is useful for extrapolation of TMTV.

## 4 DISCUSSION

Soluble IL‐2R is a soluble form of the α‐subunit of high‐affinity receptor for IL‐2 that consists of three subunits: α‐subunit, β‐subunit, and γ‐subunit. While resting lymphocytes, monocytes, and NK cells constitutively express the β‐ and γ‐subunits, the α ‐subunit of IL‐2R (IL‐2Rα) is constitutively expressed only on the cell surface of lymphoid neoplastic cells and transiently induced on the activated normal lymphocytes.^25^ Although the precise mechanism of sIL‐2R shedding is not clear, it has been shown that the release of sIL‐2R is proportional to its cell surface expression, suggesting that serum levels of sIL‐2R represent the numbers of IL‐2R α‐expressing lymphoma cells and activated lymphocytes.^26^

Previous studies have shown the predictive role of pretreatment TMTV and sIL‐2R for survival in patients with FL 13, 27, 28 and DLBCL.^11^, ^15^, ^16^ Ennishi et al reported that sIL‐2R > 1000 mg/dL predicted worse OS and EFS after R‐CHOP in patients with newly diagnosed DLBCL. Goto et al reported that sIL‐2R ≥1300 U/mL predicted worse prognosis both in GCB‐like and in non‐GCB‐like DLBCL classified based on Hans criteria.^29^ The cutoff value of sIL‐2R in the current study was slightly different from those reported in previous studies.^15^, ^16^ Cutoff value of sIL‐2R in our study was determined using CLIA, while ELISA was used in Goto's report. Although it is possible that the different methods for sIL‐2R assessment could result in the different cutoff values, it could be also possible that the different endpoints used in ROC analyses performed by Goto et al and us resulted in the difference of cutoff values between these two studies; Goto et al used the median progression‐free survivals (PFS) as the endpoint, while we used EFS24. Ennishi et al arbitrarily used the median value of serum levels of sIL‐2R as the cutoff value.

We have extended these previous findings on the prognostic values of TMTV and serum levels of sIL‐2R at diagnosis. The primary aim of this study was to clarify the correlation between serum levels of sIL‐2R and TMTV and compare the role of these factors as prognostic biomarkers. We found significant positive correlation between serum levels of sIL‐2R and TMTV. Furthermore, sIL‐2R ≥1300 U/mL stratified patients with poor prognosis in an analogous manner to TMTV ≥150 cm^3^ did. These cutoff values also improved risk stratification of patients with NCCN‐IPI High and High‐Int.

Serum levels of sIL‐2R have been routinely measured in DLBCL patients since 1990s in Japan.^30^ This biomarker is also used as a major prognostic biomarker for transplant‐related GVHD in United States.^31^ Even though we have 4.3 PET units per 1 million people in Japan (3rd in the world), some hematology/oncology centers are not equipped with PET, and patients need to travel to the external PET facilities (OECD stat. 2017 https://stats.oecd.org/index.aspx?DataSetCode=HEALTH_STAT#). Instead, we can know the serum levels of sIL‐2R within a day of blood sampling in many centers. In Japan, the costs associated with ^18^FDG‐PET and sIL‐2R are ~1000 and 40 USD, respectively. Thus, serum level of sIL‐2R is a promising biomarker that can be easily and inexpensively measured in clinical practice and have a great potential as a predictor of outcome in DLBCL patients; however, it should be noted that PET/CT is also useful for monitoring of tumor responses after treatment, suggesting that these two tests could work together in a complementary fashion. It was reported that profound reduction of TMTV from initial PET/CT to interim PET‐CT was associated with better prognosis, indicating that TMTV on interim PET/CT could be the useful biomarker in DLBCL*.* 32 Further studies are required to determine whether serum levels of sIL‐2R after treatment could be correlated with TMTV on interim PET/CT.

The accurate evaluation of tumor burden at diagnosis became more important in the rituximab era, because high tumor amounts promote clearance of rituximab from the circulation both in mice and in humans; higher TMTV at diagnosis of DLBCL led to lower rituximab exposure and inferior OS and PFS,^33^ suggesting that rituximab dosing could be guided by tumor amount at diagnosis. Metavol^®^ is a free and open‐source software tool to measure TMTV from PET/CT scans. Although this software made measuring TMTV much easier, our data indicate that sIL‐2R also correlates with tumor burden and enables us to evaluate tumor burden in patients, in whom PET/CT evaluation is not available. It should be noted that serum level of sIL‐2R cannot function as all the same to PET/CT scan does, such as visualization the distribution of the tumor lesions in patients.

Our study has some limitations, including a retrospective setting, small sample size, rather low AUC in ROC analyses, diagnosis according to the previous 2008 WHO classification, lack of central review for pathological diagnosis, and use of R‐CHOP‐like chemotherapies in some patients. However, OS rate of 53.1% and EFS 45.4% at 5 years were consistent with previous studies, in which aged DLBCL patients were treated with similar regimens used in our study.^23^, ^24^, ^34^, ^35^ The difference in sIL‐2R measurement between the training and validation cohorts might impact our results; CLIA was used in the training cohort, while ELISA was used in the validation cohort. Although the upper normal limits of sIL‐2R in these two assays were similar (496 U/mL for CLIA and 500 U/mL for ELISA), it might be possible that serum sIL‐2R levels differ slightly with methods of measurement kits. At least, the cutoff value of sIL‐2R determined in the training cohort successfully stratified the outcome of the patients in the validation cohort. The correlation between CLIA‐based and ELISA‐based levels of sIL‐2R needs to be clarified in the future studies. Another limitation of the current study is that the cameras and equipments used for PET/CT imaging were different from those used in the validation cohort.

In summary, we for the first time showed positive correlation between the serum level of sIL‐2R and the quantitative parameter TMTV in patients with newly diagnosed DLBCL. sIL‐2R is easily measurable in the clinical practice and have a great potential to predict treatment outcomes and assess metabolic tumor burden of DLBCL patients.

## CONFLICT OF INTEREST

The authors have no conflicts of interest to declare.

## References

[1] Morton LM , Wang SS , Devesa SS , Hartge P , Weisenburger DD , Linet MS . Lymphoma incidence patterns by WHO subtype in the United States, 1992–2001. Blood. 2006;107(1):265‐276. 16150940 10.1182/blood-2005-06-2508 PMC1895348

[2] Pfreundschuh M . How I treat elderly patients with diffuse large B‐cell lymphoma. Blood. 2010;116(24):5103‐5110. 20805363 10.1182/blood-2010-07-259333

[3] Sehn LH , Berry B , Chhanabhai M , Fitzgerald C , Gill K , Hoskins P , et al. The revised International Prognostic Index (R‐IPI) is a better predictor of outcome than the standard IPI for patients with diffuse large B‐cell lymphoma treated with R‐CHOP. Blood. 2007;109(5):1857‐1861. 17105812 10.1182/blood-2006-08-038257

[4] Wieringa A , Boslooper K , Hoogendoorn M , Joosten P , Beerden T , Storm H , et al. Comorbidity is an independent prognostic factor in patients with advanced‐stage diffuse large B‐cell lymphoma treated with R‐CHOP: a population‐based cohort study. Br J Haematol. 2014;165(4):489‐496. 24754632 10.1111/bjh.12765

[5] Zhou Z , Sehn LH , Rademaker AW , Gordon LI , Lacasce AS , Crosby‐Thompson A , et al. An enhanced International Prognostic Index (NCCN‐IPI) for patients with diffuse large B‐cell lymphoma treated in the rituximab era. Blood. 2014;123(6):837‐842. 24264230 10.1182/blood-2013-09-524108 PMC5527396

[6] Alizadeh AA , Eisen MB , Davis RE , Ma C , Lossos IS , Rosenwald A , et al. Distinct types of diffuse large B‐cell lymphoma identified by gene expression profiling. Nature. 2000;403(6769):503‐511. 10676951 10.1038/35000501

[7] Seki R , Ohshima K , Fujisaki T , Uike N , Kawano F , Gondo H , et al. Prognostic impact of immunohistochemical biomarkers in diffuse large B‐cell lymphoma in the rituximab era. Cancer Sci. 2009;100(10):1842‐1847. 19656156 10.1111/j.1349-7006.2009.01268.x PMC11158565

[8] Miyazaki K , Yamaguchi M , Suzuki R , Kobayashi Y , Maeshima AM , Niitsu N , et al. CD5‐positive diffuse large B‐cell lymphoma: a retrospective study in 337 patients treated by chemotherapy with or without rituximab. Ann Oncol. 2011;22(7):1601‐1607. 21199885 10.1093/annonc/mdq627

[9] Okamoto A , Yanada M , Inaguma Y , Tokuda M , Morishima S , Kanie T , et al. The prognostic significance of EBV DNA load and EBER status in diagnostic specimens from diffuse large B‐cell lymphoma patients. Hematol Oncol. 2017;35(1):87‐93. 26177728 10.1002/hon.2245

[10] Green TM , Young KH , Visco C , Xu‐Monette ZY , Orazi A , Go RS , et al. Immunohistochemical double‐hit score is a strong predictor of outcome in patients with diffuse large B‐cell lymphoma treated with rituximab plus cyclophosphamide, doxorubicin, vincristine, and prednisone. J Clin Oncol. 2012;30(28):3460‐3467. 22665537 10.1200/JCO.2011.41.4342

[11] Cottereau AS , Lanic H , Mareschal S , Meignan M , Vera P , Tilly H , et al. Molecular profile and FDG‐PET/CT total metabolic tumor volume improve risk classification at diagnosis for patients with diffuse large B‐cell lymphoma. Clin Cancer Res. 2016;22(15):3801‐3809. 26936916 10.1158/1078-0432.CCR-15-2825

[12] Cottereau AS , Lanic H , Mareschal S , Meignan M , Vera P , Tilly H , et al. Molecular profile and FDG‐PET metabolic volume at staging in DLBCL‐response. Clin Cancer Res. 2016;22(13):3414‐3415. 27371634 10.1158/1078-0432.CCR-16-0783

[13] Meignan M , Cottereau AS , Versari A , Chartier L , Dupuis J , Boussetta S , et al. Baseline metabolic tumor volume predicts outcome in high‐tumor‐burden follicular lymphoma: a pooled analysis of three multicenter studies. J Clin Oncol. 2016. 10.1200/JCO.2016.66.9440 27551111

[14] Cottereau AS , Becker S , Broussais F , Casasnovas O , Kanoun S , Roques M , et al. Prognostic value of baseline total metabolic tumor volume (TMTV0) measured on FDG‐PET/CT in patients with peripheral T‐cell lymphoma (PTCL). Ann Oncol. 2016;27(4):719‐724. 26787236 10.1093/annonc/mdw011

[15] Goto N , Tsurumi H , Goto H , Shimomura YI , Kasahara S , Hara T , et al. Serum soluble interleukin‐2 receptor (sIL‐2R) level is associated with the outcome of patients with diffuse large B cell lymphoma treated with R‐CHOP regimens. Ann Hematol. 2012;91(5):705‐714. 22183251 10.1007/s00277-011-1363-4

[16] Ennishi D , Yokoyama M , Terui Y , Asai H , Sakajiri S , Mishima Y , et al. Soluble interleukin‐2 receptor retains prognostic value in patients with diffuse large B‐cell lymphoma receiving rituximab plus CHOP (RCHOP) therapy. Ann Oncol. 2009;20(3):526‐533. 19074749 10.1093/annonc/mdn677

[17] Cheson BD , Pfistner B , Juweid ME , Gascoyne RD , Specht L , Horning SJ , et al. Revised response criteria for malignant lymphoma. J Clin Oncol. 2007;25(5):579‐586. 17242396 10.1200/JCO.2006.09.2403

[18] Kurtz DM , Green MR , Bratman SV , Scherer F , Liu CL , Kunder CA , et al. Noninvasive monitoring of diffuse large B‐cell lymphoma by immunoglobulin high‐throughput sequencing. Blood. 2015;125(24):3679‐3687. 25887775 10.1182/blood-2015-03-635169 PMC4463733

[19] Hirata K , Kobayashi K , Wong KP , Manabe O , Surmak A , Tamaki N , et al. A semi‐automated technique determining the liver standardized uptake value reference for tumor delineation in FDG PET‐CT. PLoS ONE. 2014;9(8):e105682. 25162396 10.1371/journal.pone.0105682 PMC4146536

[20] Maurer MJ , Ghesquieres H , Jais JP , Witzig TE , Haioun C , Thompson CA , et al. Event‐free survival at 24 months is a robust end point for disease‐related outcome in diffuse large B‐cell lymphoma treated with immunochemotherapy. J Clin Oncol. 2014;32(10):1066‐1073. 24550425 10.1200/JCO.2013.51.5866 PMC3965261

[21] Jakobsen LH , Bogsted M , Brown PN , Arboe B , Jorgensen J , Larsen TS , et al. Minimal loss of lifetime for patients with diffuse large B‐cell lymphoma in remission and event free 24 months after treatment: A Danish population‐based study. J Clin Oncol. 2017;35(7):778‐784. 28095160 10.1200/JCO.2016.70.0765

[22] Kanda Y . Investigation of the freely available easy‐to‐use software 'EZR' for medical statistics. Bone Marrow Transplant. 2013;48(3):452‐458. 23208313 10.1038/bmt.2012.244 PMC3590441

[23] Tsurumi H , Hara T , Goto N , Kanemura N , Kasahara S , Sawada M , et al. A phase II study of a THP‐COP regimen for the treatment of elderly patients aged 70 years or older with diffuse large B‐cell lymphoma. Hematol Oncol. 2007;25(3):107‐114. 17457949 10.1002/hon.815

[24] Tomita N , Kodama F , Tsuyama N , Sakata S , Takeuchi K , Ishibashi D , et al. Biweekly THP‐COP therapy for newly diagnosed peripheral T‐cell lymphoma patients. Hematol Oncol. 2015;33(1):9‐14. 24519501 10.1002/hon.2136

[25] Bien E , Balcerska A . Serum soluble interleukin 2 receptor alpha in human cancer of adults and children: a review. Biomarkers. 2008;13(1):1‐26. 17906988 10.1080/13547500701674063

[26] Junghans RP , Waldmann TA . Metabolism of Tac (IL2Ralpha): physiology of cell surface shedding and renal catabolism, and suppression of catabolism by antibody binding. J. Exp. Med.. 1996;183(4):1587‐1602. 8666917 10.1084/jem.183.4.1587 PMC2192498

[27] Prochazka V , Papajik T , Faber E , Raida L , Kapitanova Z , Langova K , et al. Soluble interleukin‐2 receptor level predicts survival in patients with follicular lymphoma treated with cyclophosphamide, doxorubicin, vincristine and prednisone chemotherapy in the rituximab era. Leuk Lymphoma. 2014;55(7):1584‐1590. 24180329 10.3109/10428194.2013.850167

[28] Umino K , Fujiwara SI , Ikeda T , Toda Y , Ito S , Mashima K , et al. Prognostic value of the soluble interleukin‐2 receptor level after patients with follicular lymphoma achieve a response to R‐CHOP. Hematology. 2017;22(9):521‐526. 28413914 10.1080/10245332.2017.1312204

[29] Hans CP , Weisenburger DD , Greiner TC , Gascoyne RD , Delabie J , Ott G , et al. Confirmation of the molecular classification of diffuse large B‐cell lymphoma by immunohistochemistry using a tissue microarray. Blood. 2004;103(1):275‐282. 14504078 10.1182/blood-2003-05-1545

[30] Motokura T , Kobayashi Y , Fujita A , Nakamura Y , Taniguchi T , Uchimaru K , et al. Clinical significance of serial measurement of the serum levels of soluble interleukin‐2 receptor and soluble CD8 in malignant lymphoma. Leuk Lymphoma. 1995;16(3–4):355‐362. 7719243 10.3109/10428199509049776

[31] McDonald GB , Tabellini L , Storer BE , Lawler RL , Martin PJ , Hansen JA . Plasma biomarkers of acute GVHD and nonrelapse mortality: predictive value of measurements before GVHD onset and treatment. Blood. 2015;126(1):113‐120. 25987657 10.1182/blood-2015-03-636753 PMC4492194

[32] Malek E , Sendilnathan A , Yellu M , Petersen A , Fernandez‐Ulloa M , Driscoll JJ . Metabolic tumor volume on interim PET is a better predictor of outcome in diffuse large B‐cell lymphoma than semiquantitative methods. Blood Cancer J. 2015;5:e326. 26207787 10.1038/bcj.2015.51 PMC4526777

[33] Tout M , Casasnovas O , Meignan M , Lamy T , Morschhauser F , Salles G , et al. Rituximab exposure is influenced by baseline metabolic tumor volume and predicts outcome of DLBCL patients: a Lymphoma Study Association report. Blood. 2017;129(19):2616‐2623. 28251914 10.1182/blood-2016-10-744292

[34] Feugier P , Van Hoof A , Sebban C , Solal‐Celigny P , Bouabdallah R , Ferme C , et al. Long‐term results of the R‐CHOP study in the treatment of elderly patients with diffuse large B‐cell lymphoma: a study by the Groupe d'Etude des Lymphomes de l'Adulte. J Clin Oncol. 2005;23(18):4117‐4126. 15867204 10.1200/JCO.2005.09.131

[35] Pfreundschuh M , Trumper L , Osterborg A , Pettengell R , Trneny M , Imrie K , et al. CHOP‐like chemotherapy plus rituximab versus CHOP‐like chemotherapy alone in young patients with good‐prognosis diffuse large‐B‐cell lymphoma: a randomised controlled trial by the MabThera International Trial (MInT) Group. Lancet Oncol. 2006;7(5):379‐391. 16648042 10.1016/S1470-2045(06)70664-7

