# Supplementary figures and images for: Serum level of soluble interleukin‐2 receptor is positively correlated with metabolic tumor volume on 18F‐FDG PET/CT in newly diagnosed patients with diffuse large B‐cell lymphoma
Source: Cancer Med. 2019 Feb 20;8(3):953–62. doi: 10.1002/cam4.1973 (PMC6434200; doi:10.1002/cam4.1973)

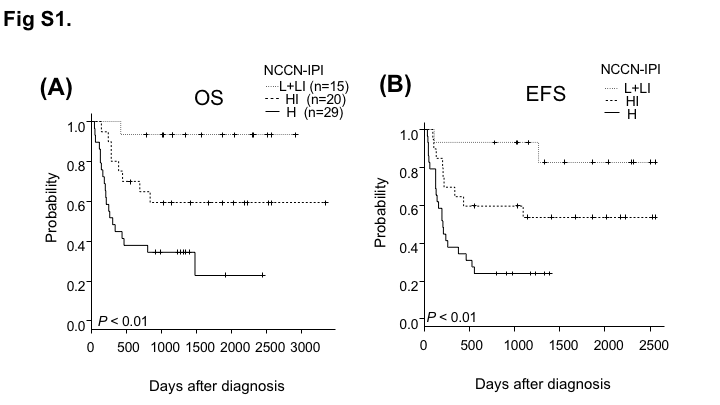

Supplement: Supplementary file 1 [file CAM4-8-953-s001.tiff]

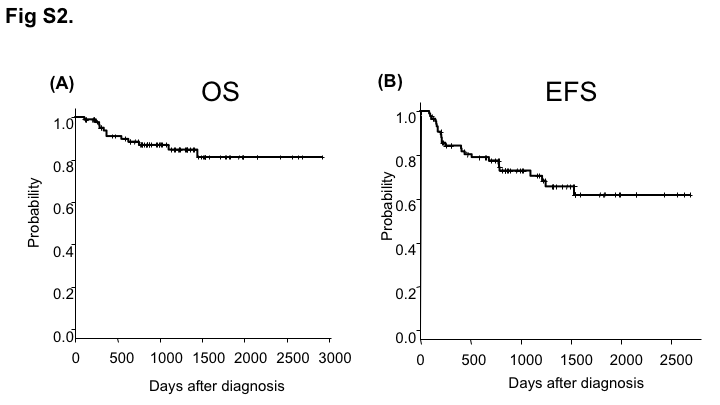

Supplement: Supplementary file 2 [file CAM4-8-953-s002.tiff]

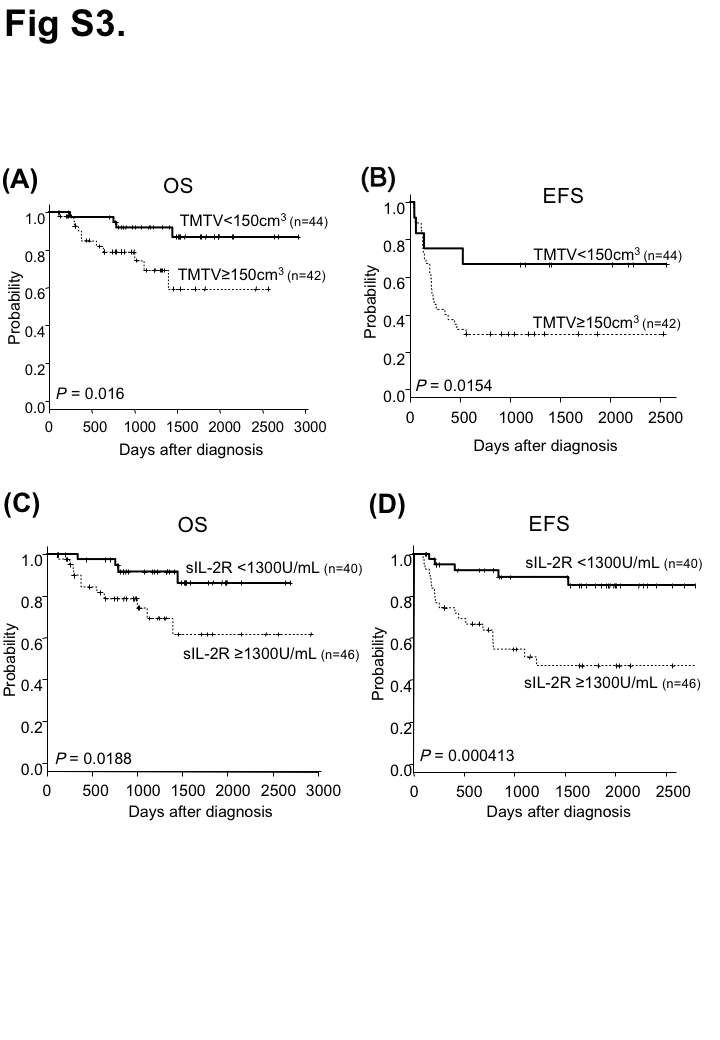

Supplement: Supplementary file 3 [file CAM4-8-953-s003.tiff]

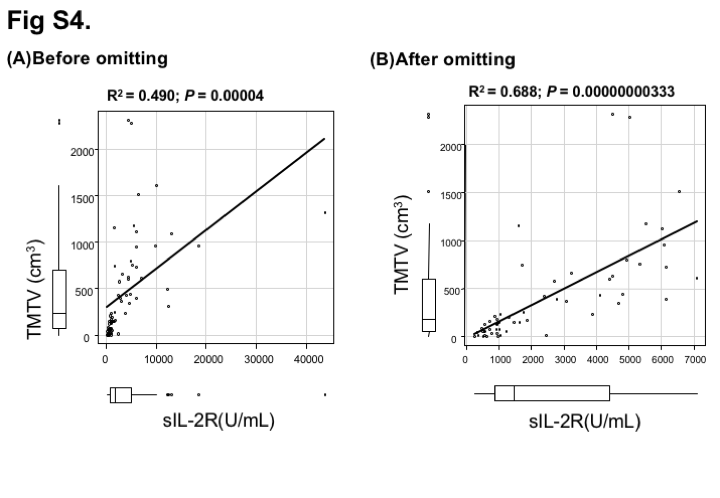

Supplement: Supplementary file 4 [file CAM4-8-953-s004.tiff]
