# Supplementary material for: Serum level of soluble interleukin‐2 receptor is positively correlated with metabolic tumor volume on 18F‐FDG PET/CT in newly diagnosed patients with diffuse large B‐cell lymphoma
Source: Cancer Med. 2019 Feb 20;8(3):953–62. doi: 10.1002/cam4.1973 (PMC6434200; doi:10.1002/cam4.1973)
Supplement: Supplementary file 2 [file CAM4-8-953-s005.docx]

Table S1. Serum levels of sIL-2R in patients with/without other poor prognostic factors in NCCN-IPI

Prognostic factors	with(IU/mL)	without(IU/nK)	*P* value
**Training cohort**			
Age ≥ 70	4119.6	3828.5	0.2[illegible]
ECOG Performance Status ≥ 2	7375.2	2622.2	<0.[illegible]
LDH > Normal	5380.6	1274.2	<0.[illegible]
Major organ involvement	4124.6	3858.7	0.1[illegible]
Stage ≥ Ⅲ	4722.1	1048.2	<0.[illegible]
**Validation cohort**			
Age ≥ 70	3771.7	2805.8	0.1[illegible]
ECOG Performance Status ≥ 2	8401.2	1985.1	<0.[illegible]
LDH > Normal	4932.5	1032.7	<0.[illegible]
Major organ involvement	4217.7	2902.3	0.[illegible]
Stage ≥ Ⅲ	6584.8	1353.5	<0.[illegible]
